# Tuning Dielectric-Magnetic Synergy in (Fe/TiC)@C Nanocomposites via Phase Composition Control for Broadband Microwave Absorption

**DOI:** 10.3390/nano16110663

**Published:** 2026-05-24

**Authors:** Nan Shen, Wenwen Wang, Jipan Zhang, Huawei Rong, Xinghao Qu, Muhammad Javid, Muhammad Farooq Saleem, Xiang Li, Muhammad Irfan, Sateesh Bandaru, Xuefeng Zhang, Gulmira Mustafayeva

**Affiliations:** 1 Zhejiang Key Laboratory of Energy Conversion Materials for Advanced Motor, Hangzhou Dianzi University, Hangzhou 310012, China; 2 School of Science, Shenyang University of Technology, Shenyang 110870, China; 3 SPIN-Lab Centre for Microscopic Research on Matter, University of Silesia in Katowice, 75 Pulku Piechoty 1A, 41-500 Chorzow, Poland; 4 Institute of Chemistry, University of Silesia in Katowice, 9 Szkolna Str., 40-007 Katowice, Poland; 5 Trier College of Sustainable Technology, Yantai University, Yantai 264006, China; 6 Kimyo International University in Tashkent, Shota Rustaveli Str. 156, Tashkent 100121, Uzbekistan

**Keywords:** Fe/TiC, nanocomposite, electromagnetic parameters, reflection loss, RCS, DFT

## Abstract

The development of cost-effective and resource-rich materials is crucial for the practical application of microwave absorbers. This study demonstrates the successful fabrication of core-shell Fe and TiC nanoparticles encapsulated within carbon shells using the arc discharge method. The samples are designated as Fe3Ti1 and Fe1Ti3, where the numbers indicate the Fe-to-Ti mass ratio in the precursor (e.g., Fe1Ti3 = 1:3 by mass). In the arc discharge synthesis mechanism, the mass ratio of Fe to Ti in the raw material was adjusted from 3:1 to 1:3 to optimize the Fe/TiC/C interfaces under a CH_4_ forming gas atmosphere. TEM analysis reveals spherical and polyhedral nanoparticles with diameters of 30–50 nm and a uniform carbon shell thickness of 3–4 nm. Raman spectroscopy shows that the Fe1Ti3 sample has a higher defect density (I_D_/I_G_ = 1.13) compared to Fe3Ti1 (0.87), indicating a more disordered carbon structure. Magnetic measurements yield saturation magnetization values of 87 emu/g for Fe3Ti1 and 50 emu/g for Fe1Ti3, with coercivities of 190.72 Oe and 203.65 Oe, respectively. When composited with paraffin at 50 wt% loading, the Fe1Ti3 sample exhibits superior microwave absorption performance, achieving a minimum reflection loss (RL) of −25.22 dB at 8.23 GHz and an effective absorption bandwidth (RL ≤ −10 dB) of 4 GHz (6.5–10.5 GHz) at a thickness of 2.5 mm. This enhanced performance is attributed to the synergistic effect of multiple loss mechanisms, including conduction loss within the three-dimensional core-shell architecture, interfacial polarization at the heterojunctions between the core and the carbon shell, and magnetic loss induced by ferromagnetic behavior associated with defects in both the shell and carbon atomic layers. The magnetic loss in the (Fe/TiC)@C nanocomposites primarily arises from the natural resonance (at ~6.5 GHz) and exchange resonance (at ~12 GHz) of the Fe cores. The dielectric loss is primarily attributed to dipole, interfacial, and space charge polarization from TiC and the carbon shell, as well as multiple scattering effects between nanoparticles. Furthermore, far-field radar cross-section simulations substantiate that the Fe/TiC@C nanocomposite demonstrates excellent radar wave attenuation capability. Further, first principles simulations reveal that introducing Fe at the C/TiC interface induces strong charge redistribution and orbital hybridization, transforming a localized dielectric interface into a highly conductive and electronically coupled C/Fe/TiC system. This interfacial modulation enhances both dielectric loss (via charge transport and polarization) and magnetic loss (via Fe-induced magnetic interactions), thereby enabling optimized dielectric-magnetic synergy for broadband microwave absorption in (Fe/TiC)@C nanocomposites.

## 1. Introduction

The proliferation of electronic devices and wireless communication technologies has intensified electromagnetic pollution, driving an urgent demand for high-performance microwave absorption materials [1,2,3,4,5]. An ideal microwave absorber should exhibit a minimum reflection loss (RL) below −10 dB (90% absorption), an effective absorption bandwidth (EAB, RL ≤ −10 dB) of at least 4–5 GHz, a density below 3 g/cm^3^, and a matching thickness typically less than 3 mm [6,7]. In recent years, carbon-encapsulated magnetic metal nanocomposites have emerged as promising candidates due to their tunable electromagnetic properties (e.g., complex permittivity ranging from 5–20 and permeability from 1–3 in the 2–18 GHz range), excellent chemical stability (oxidation resistance up to 300 °C in air), and synergistic dielectric-magnetic loss mechanisms. For instance, Fe@C nanocapsules have achieved RL values below −20 dB with EAB > 4 GHz [8,9,10]. Among these, iron-based composites are particularly attractive due to their high saturation magnetization and natural resonance characteristics [11].

However, single-component absorbers frequently exhibit limited microwave attenuation performance due to impedance mismatch and relatively narrow effective absorption bandwidths [12]. Materials based on a single loss mechanism, whether purely magnetic, dielectric, or metallic, generally cannot simultaneously balance permittivity and permeability to satisfy the impedance matching condition, which is essential for efficient electromagnetic wave absorption [12,13]. This limitation arises because the electromagnetic parameters of single-phase materials are intrinsically constrained, making it difficult to achieve the synergistic optimization of dielectric loss, magnetic loss, and impedance matching required for strong and broadband absorption [12].

TiC possesses moderate electrical conductivity (1.47 × 10^4^ S/m), which balances dielectric loss and impedance matching. In contrast, TiN exhibits a high dielectric constant leading to poor impedance matching and consequently a low reflection loss of only −16 dB. TiCN, on the other hand, requires additional carbon and nitrogen doping steps, whereas our one-step arc discharge method naturally produces TiC with controlled carbon shells. To address the limitations of single-phase absorbers, the integration of dielectric TiC with magnetic Fe nanoparticles within a carbon matrix offers a strategic approach to optimize electromagnetic parameters. The moderate conductivity of TiC, which is substantially lower than that of metallic Ti (2.08 × 10^6^ S/m), is advantageous for microwave absorption because it enables conductive loss mechanisms while minimizing the severe eddy current effects and impedance mismatch typically associated with highly conductive metals [12]. For example, TiC/carbonyl iron hybrid nanocomposites achieved a minimum reflection loss of −25.1 dB at 10.0 GHz, demonstrating the potential of TiC-based absorbers [14]. Encapsulation of TiC and Fe nanoparticles within a carbon shell serves to suppress these eddy currents while simultaneously improving impedance matching and introducing additional interfacial polarization losses [12].

Several studies have investigated Ti-based materials for microwave absorption. Multilayer TiC/epoxy composites achieved an RL of approximately −30 dB [15], while TiC@C nanocubes exhibited a minimum RL of about −29 dB [16]. Further improvements have been made using TiC nanowires [17] and by optimizing crystal morphology to enhance dielectric loss [18,19]. Titanium nitride (TiN) has also been investigated, but despite its high dielectric loss, its large dielectric constant often leads to severe impedance mismatch, resulting in unsatisfactory RL values of −27 dB or even −16.1 dB [20,21]. To overcome the impedance mismatch of TiN, titanium carbonitride (TiCN) offers a tunable alternative; carbon doping reduces the dielectric constant to optimize impedance matching while creating defects that enhance permittivity, reaching an RL of −40.1 dB [22]. However, the absence of magnetic loss in these purely dielectric systems still limits the ultimate absorption performance [12].

The carbon shell not only enhances oxidation resistance but also introduces interfacial polarization and conductive loss pathways. Encapsulation within nitrogen-doped carbon shells serves to eliminate surface eddy currents, improve chemical stability, and prevent nanoparticle agglomeration [12,23]. Recent studies on nitrogen-doped carbon nanocomposites—including Fe_3_O_4_@N-C [23], CoNi/N-CNT arrays [24], Co/N-CNT nanoplates [25], and FeNi/N-RGO aerogels [26]—have demonstrated superior RL exceeding −40 dB. These results suggest that designing dual-core architectures Fe/TiC coated with carbon shells is a highly effective strategy. Unlike the dual-core Fe/TiCN@NC structure, here Fe and TiC form separate core-shell nanoparticles [12].

In this study, we report the one-step synthesis of (Fe/TiC)@C nanocomposites via a direct current arc discharge method. By adjusting the Fe/Ti mass precursor ratio, we regulate the phase composition, microstructure, and electromagnetic properties of the composites. The synergistic effect of magnetic and dielectric loss is quantitatively controlled through the initial elemental ratios, which allows for the optimization of RL in the GHz frequency range [12,23]. The core-shell architecture, combining magnetic Fe (with potential Fe_3_C formation) with dielectric TiC embedded in a graphitic carbon layer, is designed to achieve balanced impedance matching and multidimensional loss mechanisms. This work systematically investigates the influence of composition on absorption performance to provide insights into the design of efficient, lightweight, and low-cost absorbing materials.

## 2. Experimental

### 2.1. Materials

Iron and titanium powders (purity ≥ 99.9%, 300 mesh) were purchased from Beijing Nonferrous Metals Technology Co., Ltd., Beijing, China. Methane and Ar gas were bought from the commercial supplier Hangzhou Yueyi gas Co Ltd., Hangzhou, China.

### 2.2. Synthesis of (Fe/TiC)@C Nanocomposite

**Figure 1:**
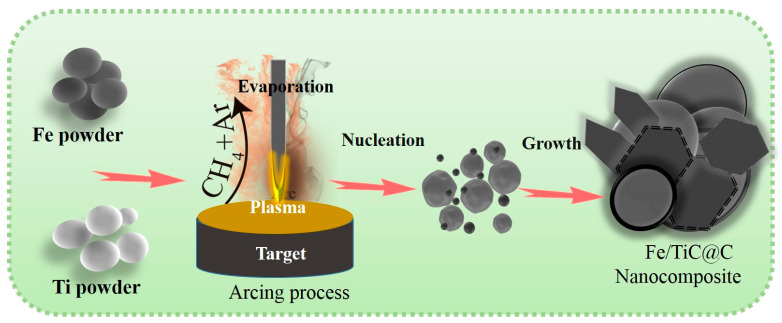
Schematic illustration of the DC arc discharge setup for the synthesis of (Fe/TiC)@C nanocomposites.

The (Fe/TiC)@C nanocomposite was synthesized via a direct current arc discharge method. A bulk Fe-Ti alloy ingot, prepared with a predetermined atomic ratio (Fe:Ti = 3:1 and 1:3), served as the anode and was placed on a water-cooled copper stage. A high-purity graphite rod (3 mm in diameter) was used as the cathode. The reaction chamber was first evacuated to a high vacuum of 10^−3^ Pa. Subsequently, a mixed atmosphere of methane (CH_4_, 0.01 MPa) and argon (Ar, 0.02 MPa) was introduced. An arc was ignited and maintained by applying a voltage of ~25 V and a current of 110 A, leading to the evaporation of the Fe-Ti alloy target. The arcing process lasted for 10 min. After completion, the arc was extinguished, and the chamber was passivated under an Ar atmosphere for 6 h to allow for controlled cooling and carbon encapsulation. The final product, a dark powder, was collected from the inner walls of the chamber for further characterization and testing. A schematic diagram of the fabrication process is shown in Figure 1.

### 2.3. Characterization

The microstructure and morphology of the nanocomposites were analyzed using Field Emission Scanning Electron Microscopy (FESEM, Hitachi, SU-5000, Tokyo, Japan) and Transmission Electron Microscopy (TEM) coupled with High-Resolution TEM and Energy Dispersive X-ray Spectroscopy (HRTEM/EDX, JEOL JEM F200, Tokyo, Japan). Phase identification was performed via X-ray Diffraction (XRD, Shimadzu XRD-6100, Kyoto, Japan) with Cu Kα radiation. The structural characteristics of the carbon component were examined using Raman spectroscopy (inVia Renishaw, Gloucestershire, UK) with a 532 nm laser at 10% intensity. The magnetic properties of the nanocomposites were measured at room temperature using a Vibrating Sample Magnetometer (VSM, Lakeshore 7400S, Westerville, OH, USA).

### 2.4. Electromagnetic Wave Parameters Measurement

To evaluate the electromagnetic wave (EMW) absorption performance, the as-synthesized (Fe/TiC)@C powder was uniformly mixed with paraffin wax in mass ratios, 50 wt%. The homogeneous mixture was then pressed into toroidal-shaped compacts with an outer diameter of 7.00 mm, an inner diameter of 3.04 mm, and a thickness of approximately 3.00 mm. The complex permittivity and permeability of these composites were measured in the frequency range of 1–18 GHz using an Agilent PNA N5224A vector network analyzer via the coaxial line method. The microwave absorption performance evaluated by *RL* was calculated based on the transmission line theory using the following equations:

(1) $$RL\left(\mathrm{dB}\right)=20log\left|\frac{Z_{in}-1}{Z_{in}+1}\right|$$

(2) $$Z_{in}=\sqrt{\frac{\mu_{r}}{\varepsilon_{r}}}tanh\left[j\left(\frac{2\pi fd}{c}\right)\sqrt{\mu_{r}\varepsilon_{r}}\right]$$

where *Z_in_* represents the input characteristic impedance, *j* is the imaginary unit, *ε_r_* and *μ_r_* are the complex permittivity (*ε_r_* = *ε*′ + *jε*″) and permeability (*μ_r_* = *μ*′ + *jμ*″) of the absorber, respectively, *f* (GHz) is the frequency, *d* (m) is the thickness of the samples, and *c* is the speed of light in free space (3 × 10^8^ m s^−1^). Paraffin wax, being largely transparent to microwaves, serves primarily as a binder to form the composite and establish a continuous absorbing matrix, allowing the intrinsic EMW attenuation properties of the (Fe/TiC)@C nanocomposite to be accurately assessed.

### 2.5. Simulation of the Radar Cross Section (RCS) of the (Fe/TiC)@C Nanocomposites

The CST Microwave Studio software 2021 was employed to conduct RCS simulations at 9.84, 11.2, and 12.56 GHz using a classic two-layer model. The upper layer was coated with various materials (a flat plate with length, width, and thickness of 180 mm, 180 mm, and 2.5 mm), while the lower layer was a perfect electric conductor (PEC, with identical dimensions: 180 mm × 180 mm × 0.5 mm). The flat plate was positioned in the X-O-Y plane, with the electromagnetic wave incident along the positive *Z*-axis under open boundary conditions.

### 2.6. DFT Simulations Methods

The calculations were performed using spin-polarized density functional theory (DFT) within the Vienna ab initio Simulation Package (VASP) [27,28], utilizing the projector augmented wave (PAW) method [29]. Exchange–correlation effects were described via the Perdew–Burke–Ernzerhof (PBE) functional under the generalized gradient approximation (GGA). A plane-wave cutoff energy of 550 eV was adopted to achieve well-converged total energies and stresses during structural optimization. The Brillouin zone was sampled with Γ-centered k-point meshes, employing a 4 × 4 × 1 grid for the C/Fe/TiC/C interface structure. A vacuum layer of approximately 15 Å was introduced along the surface-normal direction to prevent spurious interactions between periodic images. Structural relaxations proceeded until the total energy converged to within 10^−6^ eV and the residual forces on all atoms fell below 0.01 eV/Å. Dispersion interactions were incorporated through van der Waals corrections using the Grimme scheme with Becke–Johnson damping [30]. In addition, we calculated the difference between the charge densities of these structures (Δρ). The charge densities are calculated using the following equation:

(3) $$\Delta {\rho \;=\;\rho }_{\left(C/\mathrm{Fe}/\mathrm{TiC}/C\right)}\;-{\;[\rho }_{\left(C/\mathrm{Fe}\right)}{\;+\;\rho }_{(\mathrm{TiC}/C)}]$$

Here, $\rho_{\left(C/\mathrm{Fe}/\mathrm{TiC}/C\right)}$ is the total charge densities of interface; $\rho_{\left(C/\mathrm{Fe}\right)}$ and $\rho_{(\mathrm{TiC}/C)}$ are the charge densities of C/Fe and TiC/C surface slabs, respectively.

## 3. Results and Discussion

### 3.1. Microstructure Analysis

#### 3.1.1. Transmission Electron Microscopy Analysis

**Figure 2:**
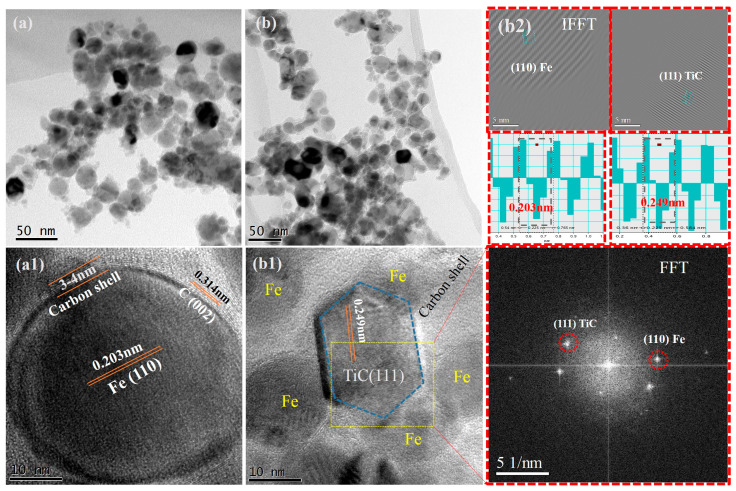
(**a**,**b**) TEM images of samples Fe3Ti1 and Fe1Ti3, respectively. (**a1**,**b1**) HRTEM images showing lattice fringes of Fe (110), TiC (111), and graphitic carbon (002), (**b2**) FFT diffractograms (fast Fourier transform) calculated from the HRTEM image revealing characteristic diffraction spots indexed to bcc Fe (110), and fcc TiC (111), and Inverse FFT (IFFT) filtered images showing enhanced lattice fringe contrast.

Figure 2 shows TEM micrographs of the Fe3Ti1 and Fe1Ti3 nanocomposites. Consistent with the XRD phase analysis, sample Fe3Ti1 consists predominantly of spherical Fe@C nanoparticles with only a small amount of TiC@C. In sample Fe1Ti3, the proportion of TiC@C increases significantly. In Figure 2a, the particles exhibit a relatively uniform spherical morphology with diameters ranging from 30 to 50 nm; only a few particles display cubic characteristics. In contrast, the micrographs in Figure 2b reveal a substantial number of rhombic nanoparticles alongside spherical ones. These geometrically distinct particles are identified as the TiC@C phase, and their abundance increases with higher Ti content in the precursor alloy. The core-shell structure observed in our (Fe/TiC)@C nanocomposites where Fe and TiC cores are encapsulated within carbon shells of 3–4 nm thickness is analogous to previously reported Co-TiC@C nanocapsules synthesized by arc discharge. In those studies, the graphitic shell thickness could be controlled by tuning the carbon source concentration [31]. Both spherical and polyhedral morphologies of TiC@C particles have been reported, with the shape variation arising from different exposed crystal facets [32]. This morphological refinement may be rationalized by the dissimilar crystal structures of Fe (bcc) and TiC (fcc). The presence of two distinct phases is believed to increase the density of nucleation sites during synthesis, which consequently suppresses particle growth and mitigates agglomeration [33].

**Figure 3:**
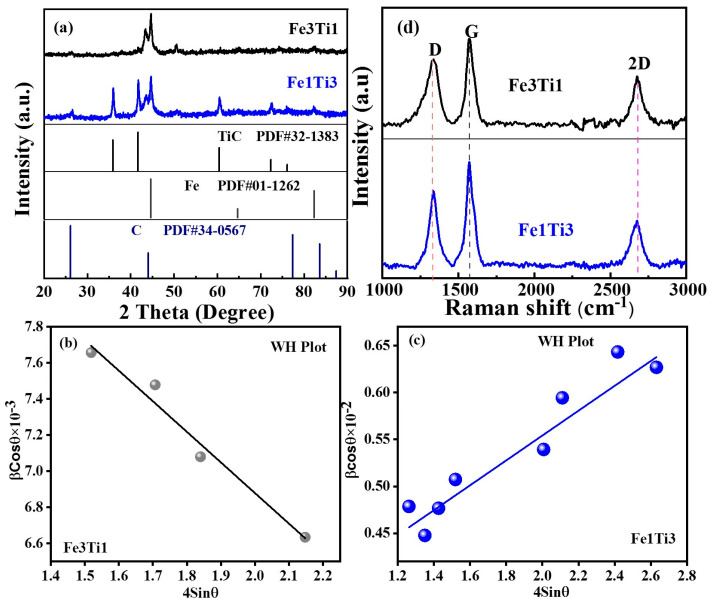
(**a**) Comparative X-ray diffraction patterns of Fe3Ti1 and Fe1Ti3, indexed to body-centered cubic Fe and face-centered cubic TiC. (**b**,**c**) Williamson–Hall plot plots for calculation of dislocation density and strains Fe3Ti1 and Fe1Ti3, respectively. (**d**) Raman spectroscopic analysis of Fe3Ti1 and Fe1Ti3, revealing D, G, and 2D bands characteristic of disordered graphitic carbon.

Figure 2a1,b1 shows the HRTEM images of both samples prepared under a mixed atmosphere of CH_4_ and Ar. Figure 2a1,b1 shows that all particle surfaces are covered with a shell-like structure, presumably a carbon atoms layer. In Figure 2a1, the lattice fringes of the shell material are clearly visible on the surface of the spherical particles observed under HRTEM. The interplanar spacing of the shell material is measured to be 0.341 nm, corresponding to the (002) crystal plane of graphitic carbon. The carbon shell thickness is relatively uniform, ranging from 3 to 4 nm. The spherical particles shown in Figure 2a1 exhibit an interplanar spacing of 0.203 nm, which corresponds to the (110) crystal plane of Fe. In Figure 2b1, most nanoparticles are spherical Fe@C, while one hexagonal particle shows an interplanar spacing of 0.249 nm, corresponding to the (111) crystal plane of TiC. To further confirm the phase identification and crystal structure, fast Fourier transform (FFT) analysis was performed on the HRTEM images using Gatan DigitalMicrograph software (version 2.01.697.0). The resulting FFT diffractograms, shown in Figure 2b2, exhibit distinct diffraction spot patterns that index to the reciprocal lattice of Fe (bcc) and TiC(fcc). For Fe3Ti1, the FFT spots correspond to the (110), (200), and (211) planes of bcc Fe, while for Fe1Ti3, additional spots corresponding to the (111), (200), and (220) planes of fcc TiC are observed. These FFT patterns are mathematically equivalent to selected area electron diffraction (SAED) patterns and provide direct crystallographic evidence consistent with the XRD analysis (Figure 3a). Specifically, the d-spacings calculated from the FFT spot positions (0.203 nm for Fe (110) and 0.249 nm for TiC (111)) match well with the XRD values. Inverse FFT (IFFT) filtering was subsequently applied to enhance lattice fringe contrast, as shown in Figure 2b2, clearly revealing the core-shell structure and the orientation relationships between the Fe and TiC crystals and their surrounding carbon shells.

#### 3.1.2. XRD Diffraction Analysis

Figure 3a presents the XRD patterns of the nanocomposites synthesized under a mixed methane and argon atmosphere with varying Fe-to-Ti mass ratios. Both samples exhibit an identical set of diffraction peaks, though with notable differences in intensity. The characteristic peaks located at 44.6°, 64.7°, and 82.3° correspond to the (110), (200), and (211) crystal planes of Fe, respectively. No diffraction peaks corresponding to any Fe-Ti alloy phase are detected, indicating that Fe and Ti did not form an alloy under the given synthesis conditions. Four distinct peaks appear at 2θ = 35.9°, 41.7°, 60.4°, and 72.4°, which can be indexed to the (111), (200), (220), and (311) planes of TiC. The formation of TiC with these specific crystal planes is consistent with previous reports on arc-discharge synthesized TiC-based nanocapsules, where similar diffraction patterns were observed for TiC cores encapsulated within graphitic shells [31,32]. The intensity of these TiC-related peaks increases markedly with higher Ti content in the precursor, confirming the enhanced formation of TiC when more Ti is available. A weak shoulder at ~45° adjacent to the Fe (110) peak corresponds to Fe_3_C (121) reflection, indicating minor iron carbide formation. The preferential formation of TiC at higher Ti content (Fe1Ti3) can be explained by three factors. First, from a thermodynamic perspective, TiC has a highly negative Gibbs free energy of formation (ΔG_f_~−180 kJ/mol at 3000 K), making it one of the most stable carbides, whereas Fe does not readily form stable carbides under arc discharge conditions. Second, kinetically, Ti atoms in the plasma have a much higher affinity for carbon than Fe atoms, leading to the reaction sequence: Ti+C→TiC (fast, exothermic) versus $Fe+C\to Fe_{3}C$ (slow, metastable). When the Ti content increases, more Ti atoms scavenge available carbon, forming TiC and leaving less carbon for Fe_3_C formation. Third, due to TiC’s much higher melting point (~3100 °C) compared to Fe (~1538 °C), TiC nuclei form earlier during condensation and grow preferentially when Ti is abundant. This competitive mechanism is evidenced by the increasing TiC peak intensity in XRD (Figure 3a) and the decreasing Fe_3_C shoulder as the Ti content rises from Fe3Ti1 to Fe1Ti3.

A comparison of the two samples reveals clear trends in phase composition. Fe is the dominant phase in Fe3Ti1, with only trace amounts of TiC. In contrast, Fe1Ti3 contains comparable quantities of Fe and TiC. A weak diffraction peak corresponding to the graphite (002) plane is observed at 26.1° in both samples, indicating the presence of graphitic carbon, which likely arises from carbon shells or free carbon particles.

The crystallite size, microstrain, and dislocation density were evaluated from the XRD patterns using both the Scherrer equation and the Williamson–Hall (W-H) method, results are presented in Table 1. The Scherrer equation, D = Kλ/(βcosθ), where K = 0.89, λ = 0.15406 nm, and β is the full width at half maximum (FWHM), yielded average crystallite sizes of 20.3 nm for Fe3Ti1 and 17.0 nm for Fe1Ti3. The W-H method, which accounts for strain broadening through the relation βcosθ = (Kλ/D) + 4εsinθ, gave crystallite sizes of 18.8 nm and 14.1 nm, respectively, with corresponding microstrain values of 1.69 × 10^−3^(Fe3Ti1) and 1.19 × 10^−3^ (Fe1Ti3). The dislocation density was calculated using ρ = 1/D^2^, yielding values of 2.31 × 10^−3^ nm^−2^ (Fe3Ti1) and 3.46 × 10^−3^ nm^−2^ (Fe1Ti3) from the Scherrer method and 5.03 × 10^−4^ nm^−2^ (Fe3Ti1) and 6.01 × 10^−4^ nm^−2^ (Fe1Ti3) from the W-H method. The smaller crystallite size and higher dislocation density in Fe1Ti3 indicate a more defective structure, consistent with the Raman analysis (I_D_/I_G_ = 1.13). The crystallinity percentage, estimated by deconvoluting the XRD patterns into crystalline peaks and an amorphous halo, was approximately 78% for Fe3Ti1 and 71% for Fe1Ti3, confirming that higher Ti content reduces long-range structural order.

#### 3.1.3. Raman Spectroscopy Analysis

In this experiment, graphitic carbon, as an important phase component, is obtained by the cracking of methane in a high-temperature environment. The carbon shell located on the outer layer of Fe and TiC particles not only improves the corrosion resistance of the nanocapsules themselves and reduces the aggregation between nanoparticles but is also an important factor affecting microwave absorption performance. Raman spectroscopy is an extremely sensitive detection method for various carbon materials.

Generally, the Raman spectrum of graphite exhibits three strong peaks, the D peak (~1350 cm^−1^), the G peak (~1580 cm^−1^), and the 2D peak (~2700 cm^−1^), while an ideal single-crystal diamond only has the D peak (~1332 cm^−1^) [34].

Figure 3b shows the Raman spectra of samples Fe3Ti1 and Fe1Ti3. The Raman spectra of the two samples are similar, with three obvious strong peaks near 1335 cm^−1^, 1572 cm^−1^, and 2672 cm^−1^, corresponding to the D peak, G peak, and 2D peak, respectively [34]. Table 2 lists the D peak and G peak parameters obtained from the fitting calculation of the Raman spectra. The D peak is redshifted relative to the generally reported D peak (~1350 cm^−1^) of graphite. This is because the graphite carbon shell in this sample was generated by rapid cooling deposition of methane cracking under non-equilibrium conditions, resulting in the destruction of symmetry in the graphite layer and severe distortion. At the same time, the half-width of the D peak increases from samples Fe3Ti1 and Fe1Ti3, which is related to the low-energy defects in graphite, generally carbon atom misalignment. The increase in the half-width of the D peak indicates that this defect is increasing. The G peak is redshifted compared to bulk graphite (~1580 cm^−1^), which may be due to the tensile stress caused by the large amount of distortion in the graphite layer. The half-width of the G peak in sample Fe3Ti1 is smaller, while that in Fe1Ti3 is basically the higher. The larger the half-width of the G peak, the lower the crystallinity of graphite. The graphitization degree of carbon in sample Fe3Ti1 is the highest. The 2D peak is the peak position that characterizes graphene. The presence of the 2D peak in both samples further confirms the presence of graphite in the samples. The broad and weak 2D peak suggests multi-layer graphitic carbon with significant disorder, consistent with the thin carbon shells observed by TEM. At the same time, there are some small sub-peaks on both sides of the main peak of the 2D peak [35,36]. Combined with the high-resolution transmission electron microscopy analysis, the thickness of the C shell is 3~4 nm, which is due to the large number of graphite layers. To a certain extent, the ratio of the intensity of the D peak to the G peak (I_D_/I_G_) can be used to evaluate the degree of graphitization. Calculations show that the I_D_/I_G_ values of sample Fe3Ti1 and Fe1Ti3 are 0.87 and 1.13, respectively, indicating that the degree of carbon ordering gradually decreases as the Ti content in the raw material increases. This decrease in graphitic ordering corresponds to an increase in structural defects within the carbon shell. While lower graphitization might appear detrimental from a structural perspective, these defects serve as polarization centers under an alternating electromagnetic field, enhancing dipole polarization and contributing positively to dielectric loss. Thus, the more disordered carbon in Fe1Ti3 is expected to improve its microwave absorption performance, compensating for its lower saturation magnetization. The increasing Ti content promotes TiC formation, which consumes carbon atoms and disrupts graphitization, leading to lower carbon ordering. At the same time, the carbon shell is relatively thick from the transmission analysis, so the cluster diameter *La* of graphite can be calculated using the Tuinstra–Koenig formula:

(4) $$La=c\times {\left(I_{D}/I_{G}\right)}^{-1}$$

The laser wavelength was 532 nm, and the constant cc is approximately 5 nm. The calculated *La* values are 5.75 nm for Fe3Ti1 and 4.42 nm for Fe1Ti3, where a lower *La* indicates more graphite defects. From Fe3Ti1 to Fe1Ti3, the graphite thickness, crystallinity, and structural order all decrease. Importantly, these defects are not mere imperfections; they enhance microwave absorption by providing polarization relaxation sites. This trade-off (lower magnetic content from less Fe versus higher dielectric loss from more defective carbon) explains the superior performance of Fe1Ti3.

### 3.2. Magnetic Analysis of Samples at Room Temperature

**Figure 4:**
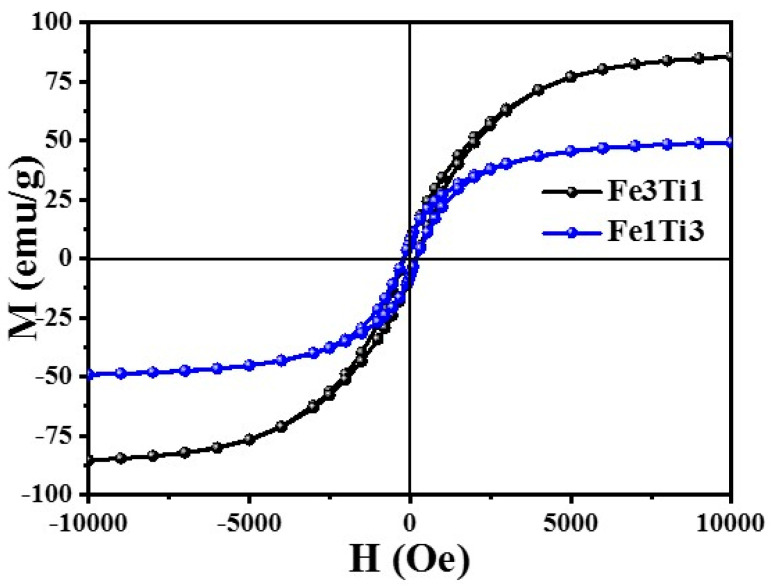
Hysteresis loops of the samples Fe3Ti1 and Fe1Ti3.

Figure 4 shows the hysteresis loops of Fe3Ti1 and Fe1Ti3 nanoparticles at room temperature, and Table 3 lists their magnetic parameters, including saturation magnetization (Ms), coercivity (Hc), and remanence (Mr). Both samples exhibit typical ferromagnetic behavior. As expected, Ms decreases with decreasing iron content: Fe3Ti1 (87 emu/g) has a higher value than Fe1Ti3 (50 emu/g). The remanence follows a similar trend, decreasing from 7.52 emu/g for Fe3Ti1 to 7.42 emu/g for Fe1Ti3. Notably, despite its lower Fe content, Fe1Ti3 exhibits a higher coercivity (203.65 Oe) compared to Fe3Ti1 (190.72 Oe). This increase is directly attributed to morphological differences between the two samples and can be explained by two factors. TEM analysis (Section 3.1.1) reveals that Fe1Ti3 has a more refined and uniform particle size distribution. Smaller Fe nanoparticles approach the single-domain size threshold (typically 20–30 nm for Fe), where magnetization reversal occurs via coherent rotation rather than domain wall motion. This reversal mechanism requires higher energy, resulting in increased coercivity. In contrast, Fe3Ti1 contains larger particles that are multidomain, where domain wall motion allows reversal at lower fields, thus reducing coercivity. The increased TiC content in Fe1Ti3 acts as a non-magnetic matrix that physically separates Fe nanoparticles. This spatial isolation reduces magnetic dipole-dipole coupling and exchange coupling between adjacent Fe particles. When particles are decoupled, each Fe nanoparticle behaves magnetically more independently, suppressing cooperative reversal and further enhancing coercivity. In Fe3Ti1, the lower TiC content means Fe particles are more closely packed, leading to stronger interparticle coupling, which facilitates magnetization reversal and lowers coercivity. The combination of lower Ms but higher Hc in Fe1Ti3 indicates that while the total magnetic moment is reduced, the material’s resistance to demagnetization is improved. The higher coercivity reflects increased magnetic anisotropy arising from the refined morphology. In the GHz frequency range, this anisotropy influences natural resonance and exchange resonance behavior rather than quasi-static hysteresis loss, contributing beneficially to microwave absorption. Thus, morphological control specifically reducing particle size toward the single-domain limit and increasing interparticle separation via a non-magnetic TiC matrix provides a direct pathway to tune coercivity and magnetic loss properties in (Fe/TiC)@C nanocomposites.

Fe1Ti3 exhibits lower saturation magnetization but higher coercivity. The increased coercivity may arise from the refined particle size and enhanced single-domain behavior of Fe nanoparticles, as the non-magnetic TiC matrix (working as a spacer) reduces magnetic coupling between Fe particles [37].

### 3.3. Microwave Absorption Performance

#### 3.3.1. Electromagnetic Parameter Analysis

**Figure 5:**
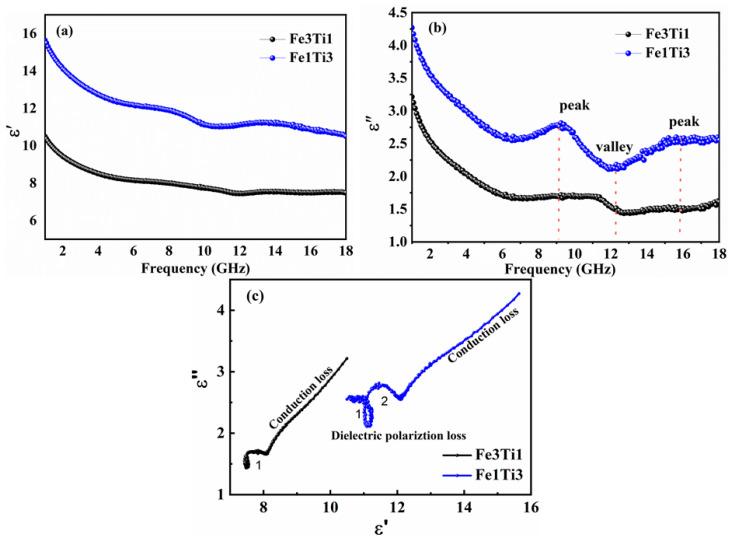
The complex permittivity of samples Fe3Ti1 and Fe1Ti3: (**a**,**b**) The real and imaginary parts of the complex permittivity, (**c**) Cole-Cole plots of samples Fe3Ti1 and Fe1Ti3.

As is well known, the real part of the complex permittivity represents the ability to store electromagnetic waves, while the imaginary part represents the ability to dissipate electromagnetic waves. As can be seen from Figure 5a, the real parts of the permittivity of samples Fe3Ti1 and Fe1Ti3 both show a trend of gradually decreasing with increasing frequency. As shown in Figure 5a, typical dielectric dispersion is observed, with the real part of the permittivity (ε′) decreasing as frequency increases. For Fe1Ti3, ε′ drops from ~16.2 at 1 GHz to ~12.9 at 18 GHz, while for Fe3Ti1, it decreases from ~10 to ~7.8 over the same range. Figure 5b shows the curves of the imaginary part of the complex permittivity of both samples as a function of frequency. It can be seen that ε″ for all samples decreases in the 1–7 GHz range and remains relatively flat from 7–18 GHz. The dielectric constant is relatively flat within the GHz range. Comparing the complex dielectric constants of both samples reveals that both the real and imaginary parts exhibit the characteristics of sample Fe3Ti1 < Fe1Ti3. As revealed from XRD and TEM characterization, samples are mainly composed of Fe, TiC, and C. The overall complex dielectric constant of the samples clearly originates from the TiC and C components. In samples Fe3Ti1 to Fe1Ti3, TiC content increases, while graphite crystallinity decreases. This reduction in graphitization introduces more defects into the carbon shell. These defects act as polarization centers, enhancing dipole polarization and contributing to the observed increase in dielectric loss. Thus, the more disordered carbon structure in Fe1Ti3 directly supports its superior microwave absorption, offsetting its lower saturation magnetization. Within the 9–12 GHz frequency band, a resonance peak exists in the imaginary part of both samples, which may be due to the increased complex dielectric loss caused by interfacial polarization between the three components.

Since electronic polarization and ionic polarization in dielectric polarization have weak responses in the microwave frequency band, the dielectric loss of (Fe/TiC)@C nanocomposites mainly includes dipole polarization, interfacial polarization and space charge polarization. To further investigate the dielectric loss mechanism, the relationship between ε′ and ε″ can be described by the Debye equation [38]:

(5) $${\left(\varepsilon^{\prime }-\frac{\varepsilon_{s}+\varepsilon_{\infty }}{2}\right)}^{2}+{\left(\varepsilon \prime \prime \right)}^{2}=\frac{1}{4}{\left(\varepsilon_{s}-\varepsilon_{\infty }\right)}^{2}$$

$\varepsilon_{s}$ and $\varepsilon_{\infty }$ are the static and optical dielectric constants, respectively. Using $\varepsilon^{\prime }$ as the *x*-axis and $\varepsilon^{\prime \prime }$ as the *y*-axis, Equation (4) can be transformed into a Cole-Cole plot, as shown in Figure 5c. In a Cole-Cole plot ($\epsilon^{\prime }$ vs. $\varepsilon^{\prime \prime }$), each semicircle represents a single Debye relaxation process, corresponding to one distinct polarization mechanism. The presence of multiple semicircles indicates that several dielectric relaxation processes contribute to the overall dielectric loss. As shown in Figure 5c, sample Fe3Ti1 exhibits one well-defined semicircle, suggesting that a single polarization mechanism, likely interfacial polarization at the Fe/C interface, dominates its dielectric response. In contrast, sample Fe1Ti3 displays three distinct semicircular arcs, revealing the coexistence of three different dielectric polarization mechanisms. These three semicircles can be attributed to different dielectric relaxation processes operating in the GHz frequency range. The primary mechanisms are as follows: (1) Dipole polarization arising from intrinsic defects in the TiC lattice and from defective sites in the carbon shell (e.g., dangling bonds, nitrogen-containing groups). These dipoles reorient under the alternating field, contributing to dielectric loss. (2) Interfacial (Maxwell–Wagner) polarization at the multiple heterojunctions present in the sample, including Fe/C, TiC/C, and Fe/TiC interfaces. The conductivity and permittivity contrasts between these phases cause charge accumulation at the interfaces, leading to relaxation in the GHz range. (3) Defect-induced polarization from localized charge carriers trapped at defect sites within the carbon shell and at core-shell boundaries. This mechanism is distinct from long-range space charge polarization, which typically occurs at much lower frequencies (Hz-kHz), and instead represents short-range charge hopping or localized relaxation at defects [38]. Furthermore, the approximately linear tail observed at the end of the Cole-Cole plots for both samples suggests the presence of conductivity loss, which arises from the migration of charge carriers within the conductive carbon network [38,39,40]. These mechanisms may originate from the following: (1) dipole polarization arises from inherent defects in TiC; (2) since the sample material contains two types of carbon-coated core-shell nanoparticles, Fe@C and TiC@C, interfacial polarization and relaxation phenomena will inevitably occur at the Fe and C and TiC and C interfaces due to the difference in electrical conductivity; (3) meanwhile, the high density of defects in the carbon shell increases the dielectric loss of the material, and the defects in the graphite layer become polarization centers, causing energy-level splitting and enhanced space charge polarization. Finally, it is worth noting that the (Fe/TiC)@C nanocomposite material dispersed in a paraffin matrix not only absorbs electromagnetic waves through the properties of the material itself when electromagnetic waves are incident, but also generates multiple scattering between particles, enhancing the microwave absorption performance [38].

The multiple dielectric relaxation processes observed in our Cole-Cole plots with three distinct arc segments in sample Fe1Ti3 are consistent with the polarization mechanisms reported for other carbon-coated magnetic metal nanocapsules. Qu et al. demonstrated that the hetero-interface between magnetic alloy cores and graphitic shells induces interfacial polarization, while defects in the carbon shell facilitate dipole polarization and conduction loss [41]. These multiple polarization mechanisms work synergistically to enhance dielectric loss. Furthermore, the incorporation of TiC introduces additional interfaces (Fe/C and TiC/C) that contribute to interfacial polarization, similar to the Co-TiC twin-core structures reported by Wang et al., where the dual dielectric phases (TiC and C) coupled with magnetic Co produced enhanced dielectric loss through multiple polarization mechanisms [31].

**Figure 6:**
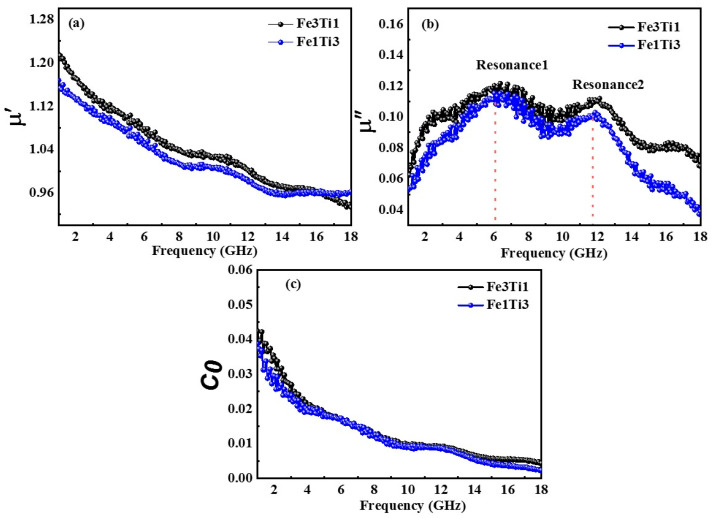
The complex permeability of samples Fe3Ti1 and Fe1Ti3: (**a**,**b**) The real and imaginary parts of the complex permeability; (**c**) Curves of the C_0_ values of the samples Fe3Ti1 and Fe1Ti3 varying with frequency.

Similar to the dielectric constant, the real part of a material’s permeability represents its magnetic polarization capability, while the imaginary part characterizes its magnetic storage capability. The magnitude of the permeability of an absorber directly affects its performance. Generally, the higher the permeability, the stronger the electromagnetic loss capability of the absorber, while also tending towards a thinner profile. Figure 6a,b show the curves of the real and imaginary parts of the complex permeability of samples Fe3Ti1 and Fe1Ti3 as a function of frequency, respectively. The real part of the complex permeability of samples shows a decreasing trend throughout the entire frequency band, with values decreasing from the initial frequency of 1GHZ. The values of permeability were reduced from 1.23 and 1.13 GHz to a cutoff frequency of 18 GHz. The imaginary parts of the complex permeability at 1GHz are 0.06, and 0.05. These values show the same trend as the frequency increases, indicating broadly similar magnetic loss mechanisms, though their magnitudes differ due to variations in Fe content and microstructure. TiC in both samples Fe3Ti1 and Fe1Ti3 is typically non-magnetic, and magnetic character is wholly provided by Fe particles. Figure 6a,b shows that the real and imaginary parts of the permeability in sample Fe1Ti3 are weaker than those in Fe3Ti1, which contains more Fe. As mentioned in the previous analysis of room-temperature static magnetism, this may be due to high-energy defects and the enhanced single-domain effect of Fe nanoparticles [37].

As shown in Figure 6b, the imaginary part of the permeability of both samples exhibits two resonance peaks at 6.5 GHz and 12 GHz. Magnetic losses in magnetic materials generally consist of eddy current losses, hysteresis losses, ferromagnetic resonance, domain wall resonance, magnetic aftereffect losses, and exchange resonance [42,43]. Hysteresis losses and magnetic aftereffect losses typically only occur in low-frequency, strong magnetic fields and can be ignored in microwave absorption; domain wall resonance occurs in low-frequency and multi-domain materials, but since Fe nanoparticles are close to a single-domain structure, they can be excluded. Therefore, the magnetic losses of the (Fe/TiC)@C nanocomposite material may originate from eddy current losses, exchange resonance, and natural resonance in ferromagnetic resonance [43]. Eddy current losses (*C*_0_) generally occur in the high-frequency range (KHz-GHz) and can be expressed according to Maxwell’s equations as follows:

(6) $$C_{0}=\frac{\mu \prime \prime }{{\left(\mu \prime \right)}^{2}f}=\frac{2}{3}\pi \mu_{0}\sigma d^{2}$$

If magnetic loss were solely due to eddy currents, the value of *C*_0_ (where *C*_0_ = *μ*″ (*μ*′)^−2^*f*^−1^) would be constant with frequency. As shown in Figure 6c, *C*_0_ varies significantly across the frequency range, indicating that eddy current loss is not the dominant mechanism and that other losses, such as natural and exchange resonance, are primarily responsible. The weak eddy current loss is attributed to the insulating carbon shell and the nanoscale particle size, which are below the skin depth, thereby suppressing eddy currents. Therefore, it can be considered that the weak eddy current loss in the (Fe/TiC)@C nanocomposite material does not play a dominant role. It can be determined that the magnetic loss mechanism of this nanocomposite is mainly natural resonance and exchange resonance. Acher et al. found through systematic research that Fe and other magnetic nanoparticles have multiple resonance peaks in the 0.1~18 GHz frequency band. The low frequency peak is the natural resonance peak, and the high frequency peak is the exchange resonance peak. It can be seen that the resonance peaks at 6.5 GHz and 12 GHz are natural resonance and exchange resonance, respectively. Exchange resonance occurs in nanoparticles smaller than the exchange length (~10–20 nm for Fe). The refined particle size in Fe1Ti3 (~30–50 nm with some smaller particles) enables this higher-order resonance mode. The magnetic loss mechanisms identified in our (Fe/TiC)@C nanocomposites, natural resonance at lower frequencies and exchange resonance at higher frequencies are characteristic of magnetic metal nanoparticles and have been similarly observed in carbon-coated FeNi alloy nanocapsules, where multiple magnetic resonances contributed to optimized impedance matching [41].

**Figure 7:**
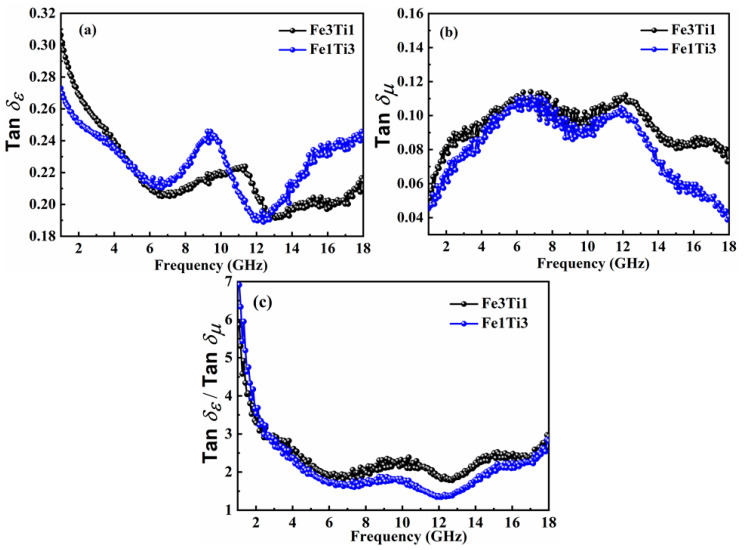
(**a**,**b**) The dielectric loss factors and magnetic loss factors of samples Fe3Ti1 and Fe1Ti3 vary with frequency. (**c**) The impedance matching degree of the two samples as a function of frequency.

Figure 7a,b shows the dielectric loss factor and magnetic loss factor of samples Fe3Ti1 and Fe1Ti3 as a function of frequency. It can be seen that sample Fe1Ti3 has the strongest dielectric loss in the frequency range of 3–18 GHz, and only in the frequency range of 1–3 GHz does the dielectric loss of sample Fe3Ti1 exceed that of Fe1Ti3. The magnetic loss mechanisms of both samples have natural resonance and exchange resonance; therefore, the magnetic loss reaches its peak at 6.5 GHz and 12 GHz and is lower at non-resonant frequencies. Meanwhile, the dielectric loss and magnetic loss of sample Fe1Ti3 are relatively similar, which helps to enhance the impedance matching of the material and improve its absorption performance.

#### 3.3.2. Reflection Loss Calculation

Impedance matching determines how much incident electromagnetic wave enters the absorber rather than being reflected at the surface. The normalized input impedance (Z_in_/Z_0_) of a metal-backed absorber is given by above Equation (2).

Perfect impedance matching occurs when |Z_in_/Z_0_| = 1, meaning the absorber’s input impedance matches that of free space (Z_0_ = 377 Ω). Deviation from unity indicates impedance mismatch and increased surface reflection.

The condition for eliminating electromagnetic wave reflection at a dielectric interface is the impedance matching between the dielectric medium and the incident medium (e.g., air)., i.e., Z_in_ = Z_0_, where the air impedance Z_0_ = 377 Ω. In this case, the electromagnetic wave experiences zero reflection at the interface and enters entirely into the dielectric. The input impedance of the material can be calculated using formula given in the experimental portion. However, in actual calculations, it can be found that in $\tanh i\frac{2\pi f}{c}\sqrt{\mu_{r}\epsilon_{r}}d$ after substituting the electromagnetic parameters, the value is always close to 1. Therefore, formula 1.10 can be simplified. We only need $\sqrt{\frac{\mu_{r}}{\epsilon \;r}}\;$ to make the value equal to 1 to achieve Z_in_ = 1. Further simplification leads to the following:

(7) $$\tan\delta_{\varepsilon }=\varepsilon \prime \prime /\varepsilon \prime =\tan\delta_{\mu }=\mu \prime \prime /\mu \prime$$

As long as formula 4 holds, Z_in =_ 1 can be obtained. Therefore, the dielectric loss tangent divided by the magnetic loss tangent can be used as an indicator of the material impedance matching degree [44]. Figure 7c shows the impedance matching degree of the two samples as a function of frequency. It can be seen that at lower frequencies (1–4 GHz), the dielectric loss of the material is strong, while the magnetic loss is weak, resulting in poor matching. Between 4–14 GHz, due to natural resonance and exchange resonances, the magnetic loss of the material increases, while the dielectric loss decreases, resulting in better matching. Between 14–18 GHz, sample Fe1Ti3 experiences a significant increase in dielectric loss and a small increase in magnetic loss, leading to a sharp deterioration in matching. Comparing the two samples, it can be seen that sample Fe3Ti1 has the best impedance matching.

Figure 7c shows the calculated |Z_in_/Z_0_| values as a function of frequency for both samples at a thickness of 2.5 mm. For Fe3Ti1, |Z_in_/Z_0_| deviates significantly from unity across most of the frequency range, indicating poor impedance matching. In contrast, Fe1Ti3 exhibits |Z_in_/Z_0_| values closer to 1, particularly in the 6–12 GHz region, demonstrating better matching between the material and free space.

To further quantify the matching quality, we employ the delta (Δ) function method, which provides a rigorous criterion for impedance matching [13]. The Δ value is calculated as follows:

(8) $$\Delta \left|Sinh^{2}\left(kfd\right)-M\right|\;$$

(9) $$k=\frac{4\pi }{c}\sqrt{\epsilon^{\prime }\mu^{\prime }}\frac{Sin\left(\frac{\delta_{E}+\delta_{M}}{2}\right)}{\sqrt{Cos\delta_{E}Cos\delta_{M}}}$$

(10) $$M=\frac{4\epsilon^{\prime }Cos\left(\delta_{E}\right)\mu^{\prime }Cos\left(\delta_{M}\right)}{{\left[\mu^{\prime }Cos\left(\delta_{E}\right)-\epsilon^{\prime }Cos\left(\delta_{M}\right)\right]}^{2}(1+tan^{2}\left(\frac{\delta_{M}-\delta_{E}}{2}\right)}$$

where K and M are functions of the electromagnetic parameters [13]. Smaller Δ values indicate better impedance matching, with Δ < 0.4 generally considered acceptable for effective absorption.

**Figure 8:**
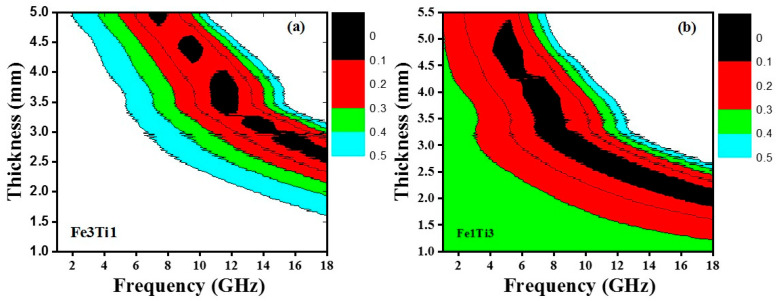
(**a**,**b**) Two-dimensional plots of characteristic input impedance matching of calculated ∆ values for Fe1Ti3 and Fe1Ti3 samples in paraffin matrix.

From Equations (7)–(9), Figure 8a,b displays the calculated ∆ plots of the three samples Fe3Ti1 and Fe1Ti3, respectively. As shown in Figure 8, Fe1Ti3 exhibits a significantly larger region with Δ < 0.4 compared to Fe3Ti1, confirming its superior impedance matching. This improved matching allows more incident waves to enter the absorber, where they can be attenuated by the multiple loss mechanisms. From the above relations, it is inferred that if the ∆ values approach zero, the commendable impedance matching will be achieved at the analogous thickness. Evidently, the region where ∆ < 0.4 is larger for Fe1Ti3 than for Fe3Ti1 which approves the relationship between *RL* and thickness values.

The superior impedance matching of Fe1Ti3 can be attributed to the balanced dielectric and magnetic properties achieved at the Fe1Ti3 ratio. The moderate conductivity of TiC and the defective carbon shell provide sufficient dielectric loss without causing excessive permittivity, while the Fe cores maintain adequate magnetic loss, together satisfying the impedance matching condition more effectively than Fe3Ti1.

The *RL* of absorbing materials is related to the material’s thickness *d*, complex permittivity, complex permeability, and the frequency *f* of the incident electromagnetic wave. Therefore, the *RL* values of (Fe/TiC)@C nanoparticle/paraffin composite materials at different thicknesses and frequencies can be calculated using the material’s electromagnetic parameters.

**Figure 9:**
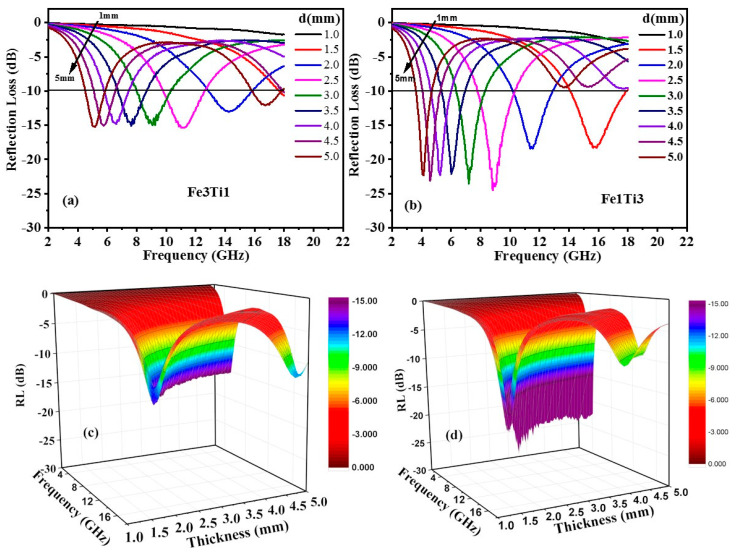
RL of (Fe/TiC)@C nanoparticles/paraffin composites: (**a**,**b**) Linear graphs, (**c**,**d**) 3D graphs of RL of both samples.

Figure 9 shows the relationship between the *RL* value and frequency for both samples. As can be seen from Figure 9, sample Fe3Ti1 has poor absorption performance; at a thickness of 2.5 mm, the minimum *RL* at 10.69 GHz is −15.43 dB. In comparison, Fe1Ti3 shows a significant improvement in *RL*, especially at a thickness of 2.5 mm. At 8.23 GHz, its RL reaches a minimum of −25.22 dB, with an effective absorption bandwidth of 6.5~10.5 GHz. It is worth noting that even at other thicknesses, the minimum *RL* of sample Fe1Ti3 is not less than −10 dB. Meanwhile, it can be observed that the absorption performance of Fe3Ti1 remains inferior to that of Fe1Ti39. Overall, sample Fe3Ti1 performs best, mainly due to its superior dielectric and magnetic losses. Furthermore, the small difference between dielectric and magnetic losses results in better electromagnetic impedance matching, thus improving microwave absorption performance.

### 3.4. Radar Cross Section (RCS) and Microwave Absorption Mechanism of Fe/TiC@C Nanocomposites

**Figure 10:**
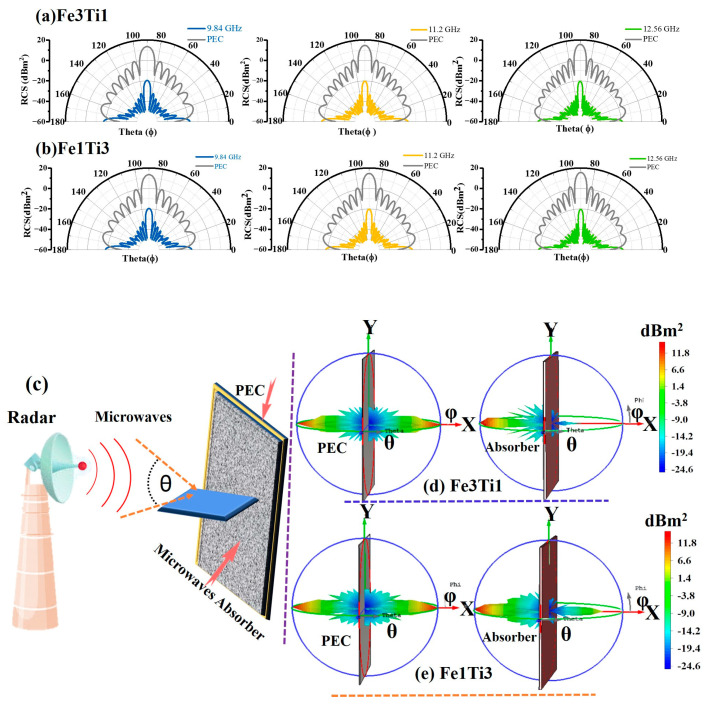
(**a**,**b**) RCS curves of (**a**) Fe3Ti1 and (**b**) Fe1Ti3 at azimuthal angles φ = −180° to +180° for 9.84, 11.2, and 12.56 GHz (thickness: 2.5 mm). The dashed line marks −10 dB·m^2^. (**c**) Far-field simulation model. (**d**,**e**) 3D radar scattering signals of bare PEC, Fe3Ti1, and Fe1Ti3 (color scale: dB·m^2^). Fe1Ti3 shows significantly reduced and diffuse scattering compared to bare PEC.

Based on the measured microwave absorption performance, frequency, and thickness of the Fe/TiC@C nanocomposites, their practical potential for far-field microwave attenuation (radar stealth) is evaluated by using CST. Figure 10a,b illustrates the RCS reduction curves of the two Fe/TiC@C nanocomposites coated PEC plates across a scanning angle range from −180° to +180° at frequencies 9.84, 11.2, and 12.56 GHz and a coating thickness of 2.5 mm.

The simulation results demonstrate that the RCS values of all nanocomposite-coated PEC plates are markedly reduced relative to the bare PEC, underscoring their robust microwave attenuation capability. Notably, the Fe1Ti3 nanocomposites achieve an average RCS value below −10 dB·m^2^ across nearly all incident angles (−180° to +180°), which is significantly better than that of the Fe3Ti1 nanocomposites. The Fe1Ti3 nanocomposites achieve a minimum RCS value of −35 dB·m^2^ in the angular ranges of −40° to −30° and +30° to +40° (Figure 10d). This exceptional performance stems from optimized impedance matching and intensified interfacial polarization, which allow most incident electromagnetic waves to penetrate the coating and be converted into heat rather than reflected. The 3D radar scattering signals of the bare PEC and FeTi-coated PEC are compared in Figure 10d. Under the same incident microwave power, the bare PEC shows an intense specular reflection signal. In contrast, the Fe3Ti1-coated PEC exhibits weakened reflection and diffuse scattering. These results confirm the potential of (Fe/TiC)@C nanocomposites for practical radar stealth applications.

**Figure 11:**
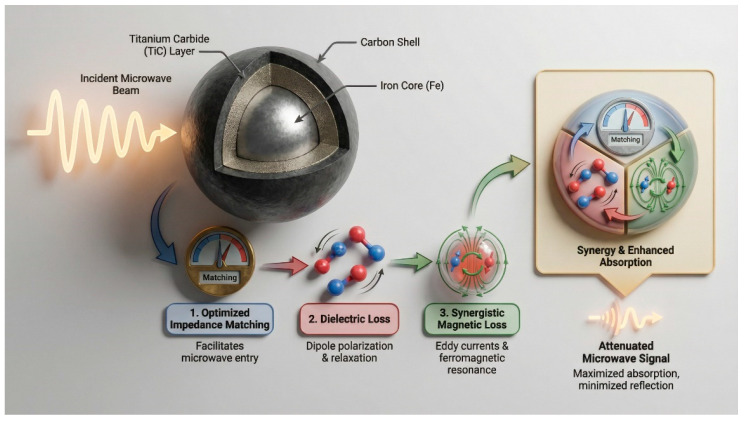
Proposed microwave absorption mechanism in the Fe/TiC@C nanocomposites.

The microwave absorption mechanisms of Fe/TiC@C arise from optimized impedance matching, dielectric loss, and magnetic loss. The enhanced performance of Fe1Ti3 (RL = −25.22 dB at 8.23 GHz) compares favorably with recent TiC-based core-shell absorbers. Wang et al. achieved exceptional absorption of in Co-TiC@C nanocapsules, which they attributed to three key factors: (i) the specific double-core shell nanostructure promoting multiple reflections and interfacial polarization, (ii) effective impedance matching between magnetic and dielectric loss components, and (iii) the synergistic effect of magnetic Co cores and dielectric TiC/C shells [31]. Similarly, in our Fe/TiC@C nanocomposites, the following mechanisms contribute to microwave absorption, as shown in Figure 11: (1) interfacial polarization at the Fe/C and TiC/C heterojunctions; (2) dipole polarization from defects in the carbon shell and TiC lattice; (3) conduction loss within the carbon shell network; (4) natural and exchange resonance from Fe cores; and (5) multiple scattering between nanoparticles. This combination of dielectric and magnetic loss mechanisms, together with optimized impedance matching achieved at the Fe1Ti3 ratio, results in the enhanced absorption performance observed for Fe1Ti3 [31,41]. First, by tailoring the Fe-to-Ti ratios to the carbon molecular structures, a balance between complex permittivity and permeability is achieved. These features result in the impedance matching ratio close to the ideal value of 1.0, suppressing the surface reflection of incident microwaves. Second, the Fe/TiC@C nanocomposites aggregated together provide electron conduction pathways and enhanced electrical conductivity, giving a moderate and highly efficient conductive loss capability for absorbing the captured microwaves. Meanwhile, the FeTi@C nanocomposites contain abundant nano-heterointerfaces, including metal-metal, metal-graphitic carbon, and graphitic carbon-amorphous carbon boundaries. This charge accumulation at heterointerfaces (Fe/C, TiC/C) under an alternating field enhances interfacial polarization loss. The carbon molecular defects act as polarization centers, inducing dipole polarization between adjacent C, leading to effective microwave absorption by increasing dipole polarization loss. This mechanism is directly supported by the Raman analysis (Section 3.1.3), which shows that Fe1Ti3 has the highest defect density (I_D_/I_G_ = 1.13, *La* = 4.42 nm). These defects enhance dipole polarization, explaining why Fe1Ti3 achieves superior absorption despite containing less magnetic Fe than Fe3Ti1. The conductive loss, interfacial polarization loss, and dipole polarization loss enhance the dielectric loss properties. Third, the FeTi cores provide substantial magnetic loss through natural and exchanges resonance effects. The microwave energy can effectively transform the synergy between these dielectric and magnetic losses into heat or other forms of energy and dissipate it.

The far-field RCS simulation results demonstrating significant radar wave reduction capability for our Fe/TiC@C nanocomposites are consistent with experimental observations from other arc-discharge synthesized core-shell absorbers, which have shown comparable potential for practical radar stealth applications [31,41].

Our work shows competitive EAB with lower Ti content, highlighting the benefit of dielectric-magnetic synergy with comparison with previous works, as shown in Table 4.

### 3.5. First Principles Simulations of (Fe/TiC)@C Nanocomposite Interface

**Figure 12:**
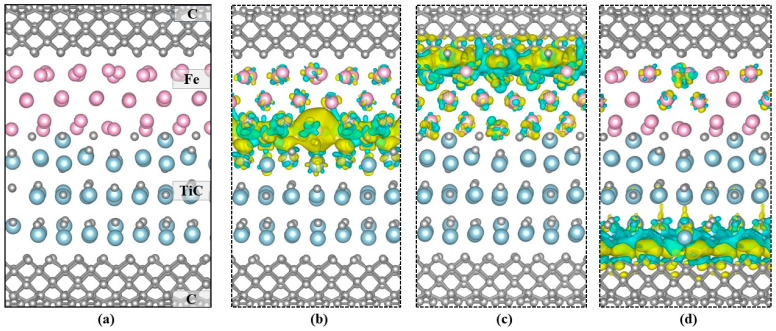
(**a**) Fully relaxed C/Fe/TiC interface: (**b**–**d**) The charge density difference at the C/Fe/TiC interface, illustrating the effect of Fe interlayer on charge transfer and bonding characteristics. Yellow and cyan isosurfaces represent charge accumulation and depletion, respectively, with the isovalue set to 0.003 e/Å^3^.

The DFT results provide a clear atomistic foundation for understanding the dielectric-magnetic synergy in (Fe/TiC)@C nanocomposites and how phase composition tuning enables broadband microwave absorption. The optimized structures reveal that the TiC slab maintains a stable surface, while the deposited carbon layer forms a relatively uniform coating above the TiC substrate. Upon introducing Fe, the interface becomes more compact, with Fe atoms occupying an intermediate position between carbon and TiC, effectively acting as a bridge that enhances interfacial contact (Figure 12a). The charge density difference results (Figure 12b–d) provide deeper insight into the interfacial electronic interactions. For the C/TiC interface, charge redistribution is primarily localized at the interface, where electron accumulation is observed around the carbon atoms and depletion near the Ti atoms. This indicates a net charge transfer from Ti to C, suggesting the formation of Ti-C bonds with mixed covalent and ionic character. However, the localization of charge implies that the interaction strength is moderate and confined to the immediate interface region.

In the C/Fe/TiC system, the charge redistribution becomes even more continuous and pronounced throughout the interface. The Fe layer clearly acts as a charge mediator, facilitating efficient electron transfer between carbon and TiC. The presence of extended regions of charge accumulation and depletion indicates strong orbital hybridization and metallic-like electron delocalization. This behavior is expected to significantly enhance electrical conductivity and interfacial stability. Overall, the results demonstrate that while the direct C/TiC interface exhibits localized bonding, the incorporation of Fe fundamentally modifies the interfacial electronic structure. The Fe interlayer strengthens bonding interactions, promotes charge delocalization, and improves electronic communication across the interface. This suggests that Fe plays a crucial role in enhancing the structural stability and functional performance of the C-coated TiC system, making it more suitable for applications requiring efficient charge transport and robust interfacial properties.

From a phase composition perspective, the DFT results suggest that controlling the relative proportions and spatial distribution of Fe, TiC, and carbon is critical. A system dominated by TiC@C will mainly exhibit dielectric behavior with limited loss efficiency, whereas incorporating an optimal amount of Fe creates multiple heterogeneous interfaces (C/Fe, Fe/TiC, and C/TiC). These interfaces act as polarization centers and facilitate multiple scattering and attenuation of electromagnetic waves. At the same time, the conductive network formed via Fe ensures that dielectric loss is neither too weak nor excessively strong, thereby achieving good impedance matching. Overall, the DFT insights reveal that Fe is not merely an additive phase but a key regulator of interfacial electronic structure. By promoting charge delocalization and magnetic coupling, Fe enables the transition from single dielectric loss to a balanced dielectric-magnetic loss system. This explains how phase composition tuning in (Fe/TiC)@C nanocomposites can achieve efficient broadband microwave absorption through optimized impedance matching, enhanced attenuation capability, and synergistic loss mechanisms.

## 4. Conclusions

This study demonstrates the controlled synthesis and structure-dependent electromagnetic performance of (Fe/TiC)@C nanocomposites prepared via DC arc discharge. Precursor composition directly governs phase formation, as varying the Fe-to-Ti mass ratio selectively tunes the relative content of spherical Fe@C and polyhedral TiC@C nanoparticles (30–50 nm), each encapsulated by a 3–5 nm carbon shell with coexisting crystalline and amorphous regions. Sample Fe1Ti3 exhibits optimized magnetic properties due to refined particle size and enhanced single-domain behavior of Fe nanoparticles. Superior microwave absorption is achieved in Fe1Ti3 with a minimum reflection loss of −25.22 dB at 8.23 GHz and an effective absorption bandwidth of 4 GHz at a thickness of 2.5 mm, attributable to three factors: enhanced dielectric loss from defect-induced dipole polarization in the disordered carbon shell (I_D_/I_G_ = 1.13, *La* = 4.42 nm), interfacial polarization at Fe/C and TiC/C heterojunctions, and magnetic resonance losses (natural and exchange resonance) from Fe nanoparticles. Notably, the increased carbon defects in Fe1Ti3 compensate for its lower saturation magnetization (50 emu/g versus 87 emu/g for Fe3Ti1), demonstrating that controlled disorder can be beneficial for microwave absorption. Compared with other TiC-based absorbers, the performance of Fe1Ti3 is competitive with Co-TiC@C (RL = −28 dB) [31] and TiC@C nanocubes (RL ≈ −29 dB), while significantly outperforming TiN-based absorbers (RL = −16 to −27 dB) due to superior impedance matching arising from TiC’s moderate conductivity. Although TiCN can achieve a higher reflection loss of −40.1 dB, it requires additional doping steps, whereas the present one-step arc discharge method produces high-performance absorbers directly using earth-abundant Fe. Based on these findings, several future research directions warrant investigation, including extending this strategy to other transition metals (e.g., Co, Ni) to further enhance magnetic loss, incorporating nitrogen doping into the carbon shell to increase defect density and polarization loss, developing three-dimensional porous architectures to increase surface area and multiple scattering, and systematically optimizing carbon shell thickness and graphitization degree to further balance dielectric and magnetic losses. Overall, this work establishes that nanocomposites with tuned phase ratios, refined particle morphology, and hybrid carbon microstructures enable synergistic electromagnetic properties. This tunability, achieved simply by controlling the Fe-to-Ti ratio, allows precise regulation of the relative contributions of magnetic Fe and dielectric TiC to the overall absorption, offering a rational and scalable design strategy for high-performance, cost-effective microwave absorbers.

## Figures and Tables

**Table 1:** Structural parameters of Fe3Ti1 and Fe1Ti3 derived from XRD analysis using the Scherrer equation and Williamson–Hall (W-H) method.

Parameters	Fe3Ti1	Fe1Ti3
Crystallite size—Scherrer (D)	20.3 nm	17.0 nm
Crystallite size—W-H (D)	18.8 nm	14.1 nm
Microstrain (ε) from W-H	1.69 × 10^−3^	1.19 × 10^−3^
Dislocation density (ρ) from Scherrer	2.31 × 10^−3^ nm^−2^	3.46 × 10^−3^ nm^−2^
Dislocation density (ρ) from W-H	5.03 × 10^−4^ nm^−2^	6.01 × 10^−4^ nm^−2^

**Table 2:** Raman components of the samples Fe3Ti1 and Fe1Ti3.

	D Peak		G Peak		Fitting Calculation	
Sample Number	Peak (cm^−1^)	Half Peak Width (cm^−1^)	Peak (cm^−1^)	Half-Peak Width (cm^−1^)	I_D_/I_G_	*La* (nm)
Fe3Ti1	1335	68.6	1570	53.4	0.87	5.75
Fe1Ti3	1335	100.1	1572	61.3	1.13	4.42

**Table 3:** Magnetic properties of samples Fe3Ti1 and Fe1Ti3 at room temperature.

Samples	Saturation Magnetization (emu/g)	Coercivity (Oe)	Remanence (emu/g)
Fe3Ti1	87	190.72	7.52
Fe1Ti3	50	203.65	7.42

**Table 4:** Comparison of microwave absorption performance with literature.

Material	RLmin (dB)	EAB (GHz)	Thickness (mm)	Ref.
Fe1Ti3@C	−25.22	4.0 (6.5–10.5)	2.5	This work
Co-TiC@C	−28.0	~3.5	2.0	[31]
TiC/epoxy	−30	~2.0	2.0	[15]
TiC@C	−29	~3.0	2.0	[16]
TiCN	−40.1	~3.5	1.8	[22]
C@TiC	−35.64	~4.0	2.0	[32]
FeNi@C	−32.5	4.2	2.0	[41]

## Data Availability

The data that support the findings of this study are available from the corresponding authors upon request.
